# Erythema Multiforme Associated With Phenytoin and Cranial RadiationTherapy (EMPACT) Syndrome Associated With Cranial Radiotherapy and Levetiracetam: A Case Report

**DOI:** 10.7759/cureus.21989

**Published:** 2022-02-07

**Authors:** Tugba Yilmaz, Berrin B Yavuz, Gul Kanyilmaz, Meryem Aktan, Selami A Temiz

**Affiliations:** 1 Department of Radiation Oncology, Meram Medical Faculty, Necmettin Erbakan University, Konya, TUR; 2 Department of Dermatology, Meram Medical Faculty, Necmettin Erbakan University, Konya, TUR

**Keywords:** antiepileptic drug, levetiracetam, glioblastoma, radiotherapy (rt), empact syndrome

## Abstract

Cranial radiotherapy (RT) is an irradiated way to treat patients with brain malignancies. Seizure is the most common symptom. Due to the frequency of seizure risk, cranial RT is usually received concomitant with previously initiated antiepileptic drugs (AED). This combination can lead to erythema multiforme (EM) like serious skin reactions starting from the irradiated port site and spreading to whole cutaneous surfaces and mucosal membranes. This clinical entity is named after as an acronym of components which are Erythema Multiforme associated with Phenytoin And Cranial RadiationTherapy as EMPACT syndrome. Most cases of EMPACT syndrome are reported with phenytoin in the literature, but there are no reported cases with levetiracetam to the best of our knowledge in the literature. Here, we report a 62-year-old male with glioblastoma, presented with severe conjunctivitis, extensive bleeding erosions in his oral mucosa and erythematous macular eruptions on the right temporoparietal port region of the scalp, and EM-like generalized lesions involved neck, chest, back, and arms following the end of his cranial RT. He was diagnosed with EMPACT syndrome, related to using levetiracetam concomitant with cranial RT. Early diagnosis is crucial for the complete response of treatment. Physicians should be alert to possible skin and mucosal reactions of patients under levetiracetam treatment, especially co-existing use of cranial RT.

## Introduction

Cranial radiotherapy (RT) is a treatment method of primary and metastatic intracranial malignancies with or without chemotherapy. Skin reactions associated with radiation are seen in most cancer patients receiving RT. Possible skin lesions in the port field are usually characterized by swelling, redness, pigmentation, edema, fibrosis, ulceration, pain, warmth, and itching of the skin [1]. However, extensive skin involvement such as erythema multiforme (EM), Stevens-Johnson Syndrome (SJS), and toxic epidermal necrolysis (TEN) are not caused by RT alone. One of the potential triggers is antiepileptic drugs (AED). Traditionally, AED usage has been a routine way of seizure prophylaxis. For these patients, most experts recommend levetiracetam initiation after having a seizure [2,3]. While these patients are under dual treatment for brain malignancies, there is a possible risk of RT dermatitis, anticonvulsant hypersensitivity reaction, and a rare clinical entity called EMPACT syndrome. Investigators named EMPACT syndrome as an acronym of components which are Erythema Multiforme associated with Phenytoin And Cranial RadiationTherapy. Clinical presentation of the syndrome is EM-like serious targetoid skin reactions starting from the RT port site and spreading to whole cutaneous surfaces and mucosal membranes [4].

There is not any reported case with levetiracetam as the primary cause of EMPACT syndrome, to the best of our knowledge in the literature. This case is unique with the type of AED. We have two objectives of the case. First of all, to be able to increase the awareness of the syndrome among physicians. Second, presenting that levetiracetam could be one of the triggers of the syndrome even though it has been reported as one of the safest AED with the cranial RT. This case was previously presented as a poster at the Virtual Congress of Turkish Society for Radiation Oncology on November 20-22, 2020.

## Case presentation

A 62-year-old male patient presented at the emergency service with a headache and vomiting in July 2019. Magnetic resonance imaging revealed a 3 cm solid lesion in his right temporoparietal junction. He had a gross-total resection in August 2019. He was diagnosed with glioblastoma. Four weeks after the operation, he underwent cranial RT, a total of 6000 cGy over 30 fractions concurrently with 150 mg/d temozolomide orally. After his first week of treatment, he started to use levetiracetam 500 mg orally twice daily for seizure prophylaxis. He neither had additional chronic diseases or previous skin problems nor he used additional drugs except for temozolomide and levetiracetam. He was examined and his laboratory findings were checked every week during his treatment.

**Figure 1:**
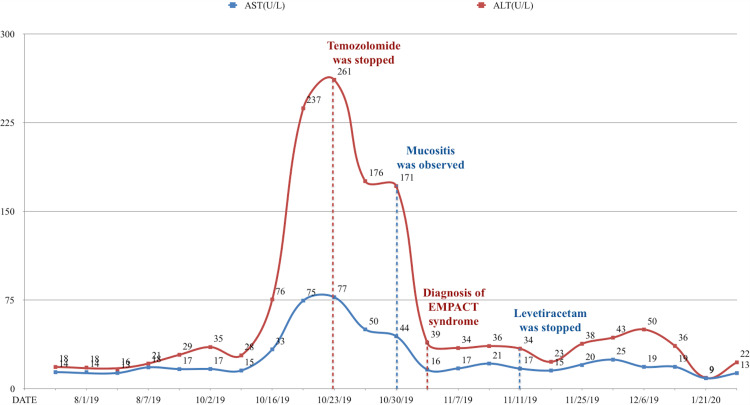
Liver function test levels and clinical findings of the patient Increasing LFT and clinical findings are seen prior to diagnosis of EMPACT syndrome. In his 26^th ^fraction of cranial RT, LFT was at the maximum level when he was not diagnosed yet. After diagnosis and treatment LFT returned back to normal values. AST: aspartate transaminase; ALT: alanine aminotransferase; RT: radiotherapy; EMPACT: Erythema Multiforme associated with Phenytoin And Cranial RadiationTherapy; LFT: liver function tests; EM: erythema multiforme

In the 26th fraction of cranial RT, his liver function tests (LFT) elevated (Figure 1). He had erythematous macular eruption, diagnosed as grade two radiation dermatitis on his port area. His temozolomide was held and he continued with cranial RT and levetiracetam on October 23, 2019. During his follow-up, viral markers and other laboratory findings were normal. However, his LFT elevation continued (Figure 1). Accompanying eosinophilia or inflammatory parameters were not detected. Erythematous macular eruption associated with moist desquamation, grade three radiation dermatitis on the port site, conjunctivitis in both eyes, and oral mucositis were detected. He had completed his 30th fraction of cranial RT and was discharged with prophylactic levetiracetam 500 mg orally twice daily.

**Figure 2:**
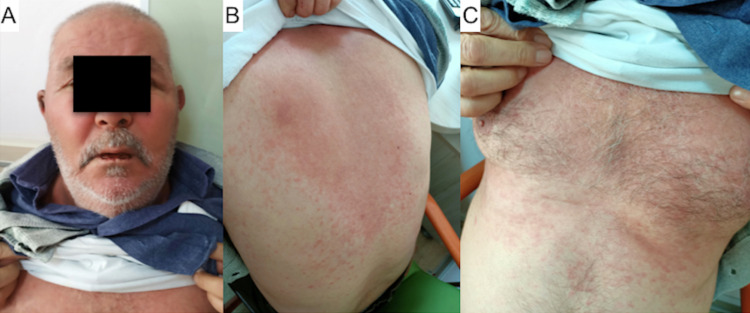
Physical exam findings prior to diagnosis of EMPACT syndrome Less than a week after completion of cranial RT patient presented with erythematous macular eruptions which tend to be common and some bullous targetoid lesions on the right temporoparietal region of the scalp, conjunctivitis in both eyes, oral mucositis (A). There are additional EM-like generalized lesions involving the neck, chest, back, and arms (B-C). RT: radiotherapy, EM: erythema multiforme; EMPACT: Erythema Multiforme associated with Phenytoin And Cranial RadiationTherapy

**Figure 3:**
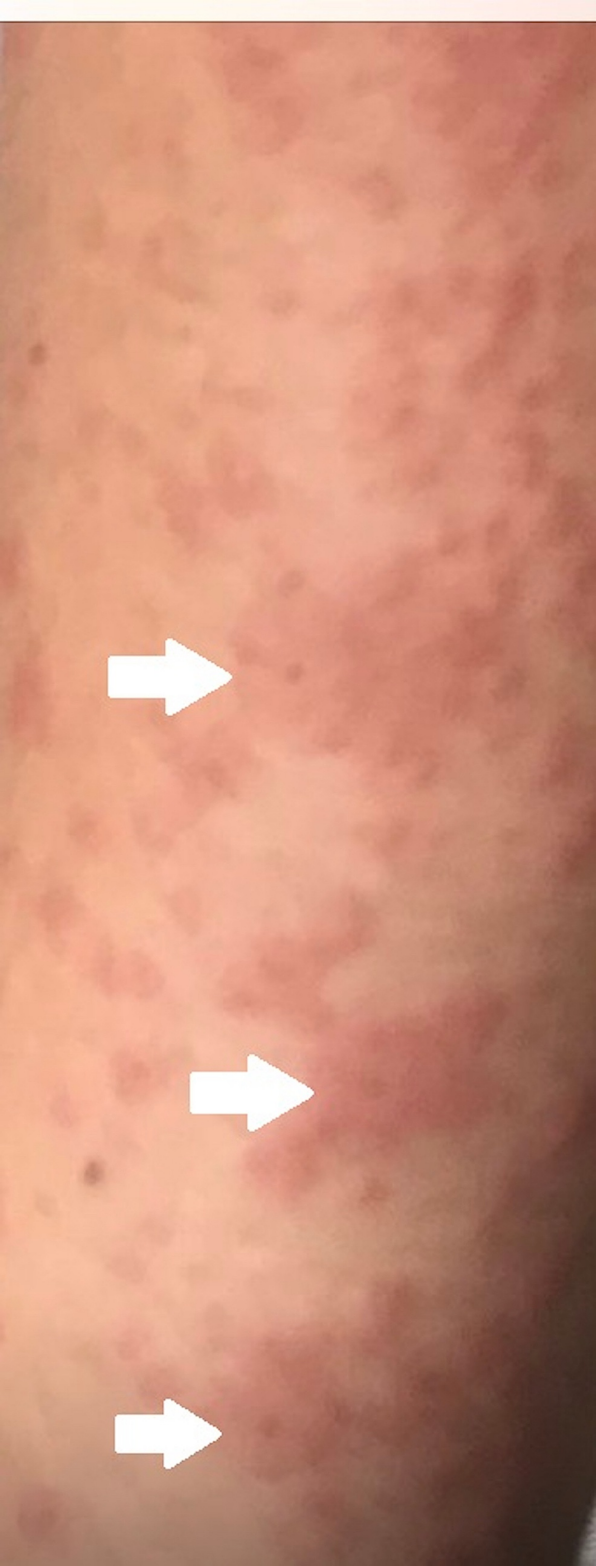
Targetoid skin lesions After dermatology consultation, typical appearance of skin lesions was diagnosed as EM and due to typical appearance biopsy was not taken. All clinical findings, only the type of AED exception, helped for the diagnosis of EMPACT syndrome [4]. EM: erythema multiforme; AED: antiepileptic drugs; EMPACT: Erythema Multiforme associated with Phenytoin And Cranial RadiationTherapy

He was admitted to our outpatient unit with severe conjunctivitis, extensive bleeding erosions in his oral mucosa on October 30, 2019 after his discharge. Erythematous macular eruptions which tend to be common and some bullous targetoid lesions on the right temporoparietal region of the scalp, and EM-like generalized lesions involving the neck, chest, back, and arms (Figure 2) were observed. After dermatology consultation, he was hospitalized with a diagnosis of EMPACT syndrome. The cutaneous lesions were typical (targetoid lesions in Figure 3), so the biopsy of lesional skin was not taken. Levetiracetam was discontinued with neurology consultation on November 11, 2019. Prednisolone 80 mg/IV was started and 25% of the dose was tapered with every clinical recovery. Eau borique dressings, topical prednisolone acetate cream, antihistaminic treatment were included. He was discharged after six weeks of hospitalization. Lesions regressed without causing any scar.

After his recovery from EMPACT syndrome, he started his adjuvant temozolomide treatment in January 2020. There was not any LFT elevation during cycles. He did not use levetiracetam or any other AED since the cessation during EMPACT syndrome. There was no history of seizure or skin reactions. In the previously resected region, recurrence was detected in his ninth cycle of temozolomide in October 2020. He had a slight left foot drop and he received a combination of bevacizumab and irinotecan. He deceased in December 2021. We had gotten the informed consent of the patient for this case.

## Discussion

The first cutaneous side effects associated with prophylactic phenytoin usage were described in the review by Rapp et al. in 1983 [5]. Initial cases about skin reactions following cranial RT and phenytoin usage were reported by Delattre et al.in 1988 and supported immunologic pathogenesis behind the combination [3]. All in all, in 2004 Ahmed et al. combined Erythema Multiforme, Phenytoin And Cranial RadiationTherapy components and named a new syndrome as EMPACT syndrome in the literature. The main reason for the syndrome is the combination of cranial RT and phenytoin, but the pathophysiology of the syndrome has not been clearly described yet [4].

Lesions started from the port site and were mostly misdiagnosed as RT dermatitis which is limited to the irradiated area. On the other hand, extensive skin lesions could be misdiagnosed as drug eruptions, SJS, TEN. These are associated with underlying infection or medication and are rarely observed with RT alone. Sequential progress of the symptoms is the key for differential diagnosis of EMPACT syndrome. In the literature, skin lesions at the cranial irradiation site is the beginning of all cases. Later, accompanying lesions spreading other parts of the body especially the upper trunk and mucositis should help to think of EMPACT syndrome as an infrequent clinical entity in differential diagnosis for patients under concurrent treatment of cranial RT and AED [4]. Lesions can occur during or after cranial RT. Sex, age, radiation dose, and AED starting time are not associated with the beginning of the syndrome. In case reports, the elevation of LFT and inflammation markers are described [6,7].

In the literature, approximately 40 cases of EMPACT syndrome have been reported. The rest of real-life cases who received AED and cranial RT are not diagnosed or reported [8]. Patients with the right diagnosis are recovered completely without other interfering clinical problems, so early diagnosis is the key to the treatment [4].

In the literature, phenytoin is the most common cause of the syndrome. Later, there are additional cases presented with different AED such as phenobarbital due to cross-reactivity of aromatic anticonvulsants. Aromatic AED induces microsomal cytochrome 450(CYP)3A and produces oxidative metabolites. An enzyme epoxide hydrolase detoxifies them. One of the pathophysiological hypotheses of the EMPACT syndrome is phenytoin-RT produce deficiency in the enzyme and may cause immune response due to metabolites [4,9,10]. In order to prevent EMPACT syndrome, reported cases mostly suggested to choose gabapentin or levetiracetam as safer AED options during cranial RT [7,11]. Levetiracetam, a non-aromatic AED is reported as non-dependent on hepatic CYP450 isoenzyme and eliminated via renal excretion [12]. However, there is a reported case about levetiracetam initiation after cessation of phenytoin as a primary cause of EMPACT syndrome, that solution was not completely resolved the symptoms and two days later patient in worse condition was admitted to the hospital [13].

Herein, we report a patient who used temozolomide and levetiracetam concurrently with cranial RT. In the literature, LFT elevation with these drugs is a rare laboratory finding [6,7]. Also, both of the drugs do not require dose arrangement in hepatic dysfunction [12,14]. In our case, the elevation of LFT, no associated eosinophilia, typical skin findings started from the port site are matched up with the literature findings of EMPACT syndrome. Interestingly, we report a case that occurred in a patient under prophylactic levetiracetam. Up to our knowledge, this is the first reported case of levetiracetam as a primary trigger of EMPACT syndrome in the literature.

## Conclusions

Physicians need to be careful about the skin lesions of patients receiving concurrent cranial RT and AED. High index of suspicion and close follow-up of these patients under AED therapy during and after cranial RT is required to establish the right diagnosis without delay. Suspected lesions should be consulted with other departments. A multidisciplinary approach to patients with EMPACT syndrome is important during diagnosis and treatment. Early diagnosis makes difference for this curable syndrome and helps patients start their adjuvant chemotherapy as soon as possible. Further reporting of case presentations and research are needed to understand the pathophysiology of the rare condition of EMPACT.
